# A bio-behavioral intervention to decrease intravaginal practices and bacterial vaginosis among HIV infected Zambian women, a randomized pilot study

**DOI:** 10.1186/s12879-017-2436-3

**Published:** 2017-05-12

**Authors:** Maria L. Alcaide, Maureen Chisembele, Emeria Malupande, Violeta J. Rodriguez, Margaret A. Fischl, Kristopher Arheart, Deborah L. Jones

**Affiliations:** 1 Department of Medicine, Division of Infectious Diseases, University of Miami Miller School of Medicine, 1120 NW 14 Street, CRB 864 (D-90A), Miami, Fl 33136 USA; 2 Department of Obstetrics and Gynecology, University of Zambia, Lusaka, Zambia; 3 University of Zambia, Lusaka, Zambia; 4 Department of Psychiatry and Behavioral Sciences, University of Miami Miller School of Medicine, Miami, USA; 5 Department of Epidemiology and Public Health, University of Miami Miller School of Medicine, Miami, USA

**Keywords:** Intravaginal practices, bacterial vaginosis, HIV shedding

## Abstract

**Background:**

Intravaginal practices (IVP) (cleansing or introducing products inside the vagina for hygiene, health or to please sexual partners) are common among women with HIV. IVP increase the risk of developing bacterial Vaginosis (BV), the most common genital infection associated with transmission of sexually transmitted infections and HIV. This study tested a pilot intervention to reduce IVP and BV in HIV infected women in Zambia.

**Methods:**

One hundred twenty-eight HIV infected women engaging in IVP were randomized to two conditions: enhanced standard of care (*n* = 70) and experimental (*n* = 58) from May 1, 2013 to February 28, 2014. All participants received a brief educational counseling session on discontinuation of IVP, and those with BV, were provided with medical treatment for BV. Women in the experimental condition received an additional group-based, culturally tailored intervention. Participants completed questionnaires assessing sexual risk factors and IVP and were assessed for BV using Nugent criteria at baseline, 6 months and 12 months.

**Results:**

At 12-month, the proportion of self-reported use of IVPs decreased in the experimental condition: soap (28% vs. 47%); cloth or a rag (19% vs. 38%); and traditional medicines (22% vs. 42%) (all *p* < 0.05)) compared with the enhanced standard of care condition. The prevalence of BV at 6 and 12 months did not differ by study condition but averaging over study condition, prevalence of BV decreased from 64.2% at baseline to 15.6% at 6 months (*p* < 0.01) and to 23.6% at 12 months (*p* = 0.15). Using an enhanced standard of care approach and an enhanced standard of care + a group intervention, IVP and BV decreased over time, but the experimental condition had greater reduction in self-reported use of IVP.

**Conclusions:**

Future studies should address interventions in communities with high burden of IVP, BV and HIV. Interventions that could be administered during routine medical care and decrease IVP and BV are needed, and should be considered part of women’s health programs.

**Trial registration number:**

NCT03134924 (retrospectively registered 21st April 2017).

**Electronic supplementary material:**

The online version of this article (doi:10.1186/s12879-017-2436-3) contains supplementary material, which is available to authorized users.

## Background

Intravaginal practice (IVP) include intravaginal cleansing (the cleaning or washing inside the vagina with fingers or other substances for the purpose of removing fluids), or intravaginal insertion (placing something inside the vagina such as powders, creams, herbs, cloth, tissue, regardless of the time they are left inside) [1, 2]. Women engage in IVP for hygiene, health or to please sexual partners. [1, 2] IVP carry deleterious consequences for women’s health as they are associated with the development of bacterial vaginosis and the acquisition of HIV infection. [3–7].

Bacterial vaginosis (BV) is the most common female genital infection and places women at risk for adverse obstetric and gynecologic outcomes (i.e., miscarriages, preterm delivery, and complications associated with gynecological surgeries). [8, 9] In addition, BV increases the risk of HIV acquisition and transmission to sexual partners and newborns. [7, 10, 11] In HIV infected women, IVP is likely responsible for the development of BV and its associated negative health consequences, and may also contribute to increases in lower genital tract inflammation, genital HIV shedding, and subsequent HIV transmission.

Zambia is a sub-Saharan country with high rates of both HIV and BV, and Zambian women living in Lusaka commonly engage in IVP. [12–16] In Zambia, IVP are culturally driven, and typically used to promote vaginal hygiene and to please male partners. [12, 13] Previous studies in sub-Saharan Africa have sought to decrease IVP in women at risk for HIV infection, but the effect of these interventions on rates of BV has been inconsistent. [17–19] In addition, previous IVP interventions have not targeted IVP as a method to decrease BV in women with HIV infection, which could have an important impact on the health of HIV infected women and on HIV transmission.

This team previously demonstrated the association of frequent IVP with BV, and evaluated the short-term effect of a bio-behavioral intervention aimed to decrease IVP and BV among HIV-infected Zambian women. [20, 21] However, many HIV risk reduction interventions have achieved behavioral change that are not sustained over time. [22, 23] This study is a pilot study designed to examine the longitudinal impact over 12 months of a culturally tailored bio-behavioral intervention to reduce IVP and BV among HIV-infected women in Zambia. This bio-behavioral intervention includes a biomedical component (prompt diagnosis and treatment of BV) and a behavioral component (behavioral intervention for IVP). In addition, we evaluate the effectiveness of our intervention in reducing lower genital inflammation and HIV shedding. It was hypothesized that participants receiving the bio-behavioral intervention would decrease the use of IVP and BV more than those receiving an enhanced standard of care condition alone. If successful, this bio-behavioral intervention could be considered for HIV infected women as a strategy to decrease BV and its associated detrimental health outcomes.

## Methods

### Ethics statement

Institutional review board (University of Miami Miller School of Medicine) and research ethics committee (University of Zambia) approvals were obtained prior to recruitment and any assessment or study related procedures. Interested study candidates were scheduled for an appointment. In a private room, the study was reviewed with study candidates in detail, and all questions were addressed prior to provision of informed consent. Informed consent was obtained from all participants prior to participating in the study.

### Study setting

The study was conducted at a community health centre in urban Lusaka, Zambia. Procedures to develop questionnaires (focus group discussion and key informant interviews) have been previously reported [13].

### Participants

Participants were women recruited from May 2013 to February 2014, living with HIV-1 infection, at least 18 years of age, receiving antiretroviral therapy (ART), engaging in intravaginal practices and vaginal intercourse with men in the month prior to enrolment, and living in the Lusaka metropolitan area. Women were excluded from the study if they were pregnant, were on hormonal contraception or had an intrauterine device (IUD) in place to avoid the potential for induced changes in inflammatory cytokines in the genital mucosa due to contraception. Participants were enrolled 7–14 days after the first day of their menses (proliferative phase of the menstrual cycle).

### Study procedures

Participants completed a baseline assessment and were then randomly assigned to one of 2 conditions: enhanced standard of care (SOC+) or SOC+ plus group intervention (experimental). A simple random allocation computer generated sequence was utilized. The study statistician did not participate in randomization and the recruiter and laboratory personnel were blinded to the condition assignment. At each visit (baseline, 6 months and 12 months) participants completed audio computer administered self-interviews (ACASI) assessing demographic, sexual risk factors, types and reasons for engaging in IVP, followed by a vaginal examination with collection of genital tract and blood samples. Assessments using ACASI were administered by the study coordinator prior to collection of genital samples. Our pilot data suggested a 22% reduction in BV in women participating in the intervention. A sample size of 64 women per group was calculated for this pilot study to provide 80% power at the two-tailed 0.05 alpha level to detect a significant moderate effect size of 0.5 in differences in BV between the groups.

### Enhanced standard of care condition

This study provided an “enhanced standard of care” (SOC+) comparison condition, consisting of a genital tract examination, collection of a vaginal swab with gram stain of vaginal secretions, diagnosis of BV using the Nugent criteria and provision of medication (oral metronidazole) within 48 h of the examination in women with Nugent score of 7–10, regardless of the presence of symptoms. Currently, the existing standard of care for treatment of a vaginal infection such as BV in Zambia consists of syndromic management; i.e., infections are treated with oral metronidazole when women present with malodorous vaginal discharge indicative of BV, without laboratory confirmatory diagnosis. In addition, at baseline, participants received an individual education session on the risk of engaging in IVP, advice to discontinue IVP, and tips for healthy vaginal hygiene, emphasizing avoiding IVP and suggesting replacing IVP by external vaginal cleansing.

### Experimental condition: SOC+ plus group intervention

The WASH (Women’s and Sexual Health) intervention included the elements of the enhanced standard of care condition and in addition, a group-based, culturally-tailored intervention to enhance the uptake of the recommendations. The group-based intervention (~10 per group) was a 45-min group session on vaginal health and healthy vaginal practices led by two trained facilitators. Facilitators utilized an intervention manual addressing risks associated with IVP, symptoms of vaginal infections, vaginal health, women’s experience with alternative methods for vaginal care, and communication with partners about vaginal health and the risks associated with IVP. The theoretical basis for the group intervention was the Information Motivation Behavioral Skills (IMB) Model, such that the underlying components of IVP (culture, partner preference, hygiene, health and sexuality factors, motivation, and behavioral skills) were addressed to provide information and motivation to promote vaginal health. The IMB model has been applied to a variety of HIV-related issues as well as in pilot interventions to decrease IVP. [19, 20, 24, 25].

### Assessments

#### Demographics, sexual risk factors and medical history

Information on demographics, sexual risk factors and medical history was collected. Questions included age, education, employment status, income, marital status, number of children, history of STIs, HIV status of sexual partner, history of exchanging sex for money, number of partners, condom use, time to HIV diagnosis, self-reported CD4 cells per millilitre.

#### Intravaginal practices

Information on intravaginal practices was collected using a culturally tailored questionnaire assessing specific products used for IVP. Questions to assess product use in the prior month utilized a dichotomous response option: product use (1 = yes, 0 = no). Products used for IVP included water alone, soap, a cloth or sponge or rag, traditional medicines given by traditional healers, herbs or flowers taken directly from the land, lemon, salt and vinegar (see Additional file 1).

#### Genital tract examination and collection of genital samples

Vaginal samples were collected by a qualified female health care professional. A vaginal speculum was inserted and a vaginal fluid sample was collected using a cotton swab and placed on a glass slide. Gram stained for Nugent scoring was performed within 24 h of collection. Two laboratory technicians were trained prior to study onset. Training included a computer based didactic session at the laboratory site provided by the principal investigator. The reading of all the slides was performed by one trained laboratory technician blinded to the participant condition assignment. In case of unclear reading, the slide was reviewed by a second technician and the chief of the laboratory until an agreement was achieved. Quality control was undertaken by reviewing 20% of the slides at the clinic laboratory by the principal investigator and revealed 100% agreement with scoring of BV.

Cervicovaginal lavage (CVL) was collected after installing 10 ml of sterile saline into the vaginal and cervical areas avoiding the os, and letting it rest for 60 s. CVL samples were transported on ice to the laboratory for analysis within 2 h of collection [26].

#### Bacterial vaginosis

BV was diagnosed using Nugent criteria (normal vaginal flora = Nugent 0–3; intermediate vaginal flora = Nugent 4–6; BV = Nugent 7–10). Diagnosis of BV was done when Nugent score was 7 or above. Abnormal vaginal flora was defined as Nugent score of 4 or above.

#### Plasma viremia and HIV-1 genital shedding

Plasma viremia and HIV-1 genital shedding were assessed by measuring plasma and CVL HIV-1 ribonucleic acid (RNA) viral load respectively using Abbott m2000^®^ RealTime Polymerase Chain Reaction (PCR) system. CVL was aliquoted into Eppendorf tubes and directly used for determining viral load. Testing was conducted at Centre for Infectious Diseases Research in Zambia (CIDRZ) laboratories. Lower limit of detection was 20 copies per millilitre.

#### Cytokine analysis

Upon reception of the CVLs at the laboratory, CVL was aliquoted into Eppendorf tubes and spun for 10 min at 1000 x g at 4C. Supernatant was then aliquoted for cytokine analysis. For each study subject, cytokine analysis was performed to quantify the levels of interleukin (IL) IL-6 and IL-8. Cytokine measures were performed using enzyme-linked immunosorbent assay (ELISA). Commercially available cytokines kits were used (Quantikine, R&D Systems, Minneapolis MN). Cytokine analysis was conducted at baseline and 6 months only.

#### Statistical analysis

Descriptive analyses were conducted to describe sociodemographic characteristics, sexual risk factors, IVP, Nugent scores, BV, detectable plasma VL and CVL HIV-1 RNA. In the case of continuous variables, sociodemographic and medical history comparisons by condition were performed using t tests or its nonparametric alternative, the Mann-Whitney U test. For comparisons of groups using categorical variables, a chi-square or Fisher’s exact test was performed.

A series of generalized estimating equations (GEE) with a binomial distribution and logit links were conducted with time, condition, and the interaction of time and condition as predictors. Given the low prevalence of some of the practices, generalized linear mixed models (generally used to handle missing data) could not be used. To assess for differences from baseline to 6-month in IL-6 and IL-8, as they are count variables, and to avoid having to transform them into a normal distribution, GEE Poisson or Negative Binomial models with log links were performed. For all models, an independent correlation structure was used. Results from GEEs were followed by least square means analyses to test for differences from baseline to 6 months and 12 month time points, and for differences in the interactions of time and condition. A Bonferroni adjustment was used for multiple comparisons. All analyses were performed using SAS 9.4 for Windows 7. A threshold of *p* < 0.05 was used to determine statistical significance.

## Results

### Participants

**Fig. 1:**
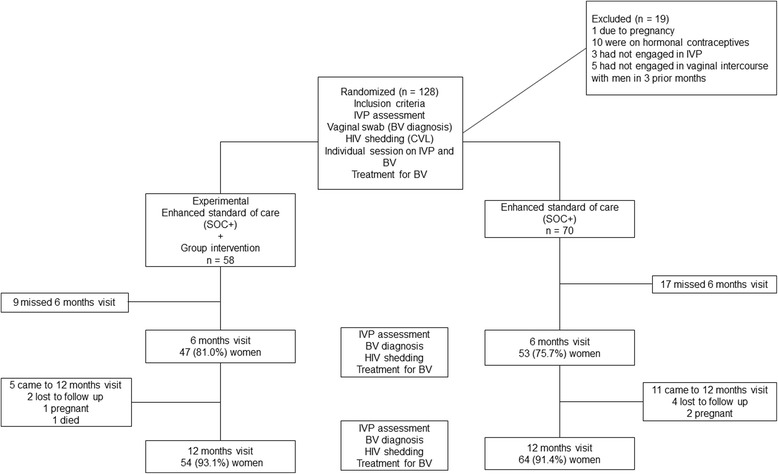
Flow diagram of women enrolled and excluded during the study period. IVP = Intravaginal Practices. BV = Bacterial Vaginosis. SOC+ = Enhanced standard of care

One hundred and twenty-eight women were enrolled and completed baseline assessments. Due to the use of simple randomization, 58 participants were enrolled into the experimental condition and 70 into the SOC+ condition. In the experimental condition 47 (81.0%) women completed the 6 month assessment and 54 (93%) completed the 12 month assessment. Four women did not complete follow-up 12 month assessments: 2 (3%) were lost to follow up, 1 (2%) became pregnant and 1 (2%) died during the study. In the SOC+ condition, 53 (76%) women completed the 6 month assessment and 64 completed the 12 month assessment (91%). Six women were did not complete follow-up 12 month assessments in the SOC+ condition: 4 (7%) women were lost to follow up and 2 (2%) became pregnant during the study. Figure 1 illustrates participation during the study.

### Demographics

Participants’ mean age was 37.3 (SD = 7.7) years and about half had completed primary school. Nearly three-fourths of the women were not working, had low income and were in a primary relationship with a male partner. Almost all had at least one child. More than three-fourths of the women reported treatment for a sexually transmitted infection (STI) in the prior year, and approximately two-thirds reporting knowing that their partner was HIV-infected. Over 90% of participants reported that they had disclosed their HIV status to their partner. About 10% of women reported having sex in exchange for some type of compensation in the prior month. Most women reported one partner in the prior 3 months and inconsistent condom use. The majority of participants had HIV infection diagnosed over 1 year ago and about one-third self-reported having CD4 counts greater than 500. Demographic characteristics and sexual risk factors did not differ significantly between those enrolled in the experimental or SOC+ conditions except that women assigned to the experimental condition less frequently reported being in a primary relationship. Detailed description of demographic characteristics and risk factors by condition can be found in Table 1.

**Table 1:** Sociodemographic and sexual risk factors characteristics of participants by condition at baseline (*n* = 128)

		Condition N(%) Median(IQR)		
	All participants	Experimental (*n* = 58)	SOC+ (*n* = 70)	*p*-value
Age	35.00 (11.00)	37.00 (14.00)	34.00 (10.00)	0.402^a^
Educational attainment				
None	8 (6.3%)	4 (6.9%)	4 (5.7%)	
Primary School	58 (45.3%)	30 (51.7%)	28 (40.0%)	
Secondary School	55 (43.0%)	21 (36.2%)	34 (48.6%)	
College/University	7 (5.5%)	3 (5.2%)	4 (5.7%)	0.514^b^
Employment status				
Work full time	23 (18.0%)	6 (10.3%)	17 (24.3%)	
Work part time	7 (5.5%)	4 (6.9%)	3 (4.3%)	
Not working	89 (69.5%)	43 (74.1%)	46 (65.7%)	
Volunteering	9 (7.0%)	5 (8.6%)	4 (5.7%)	0.206^b^
Monthly income (Zambian Kwacha)				
Less 500 (less than 65 USD)	16 (12.5%)	9 (15.5%)	7 (10.0%)	
500 to 1000 (~ 65–130 USD)	82 (64.1%)	32 (55.2%)	50 (71.4%)	
1000 to 2000 (130–260 USD)	28 (21.9%)	16 (27.6%)	12 (17.1%)	
More than 5000 (> 260 USD)	2 (1.6%)	1 (1.7%)	1 (1.4%)	0.242^b^
Marital status				
Single	25 (19.5%)	10 (17.2%)	15 (21.4%)	
Stable relationship	93 (72.7%)	39 (67.2%)	54 (77.1%)	
Unstable relationship	10 (7.8%)	9 (15.5%)	1 (1.4%)	**0.013** ^b^
Children				
No	4 (3.1%)	1 (1.7%)	3 (4.3%)	
Yes	124 (96.9%)	57 (98.3%)	67 (95.7%)	0.626^b^
Treatment of STIs in the past year (*n* = 30 women with STIs)				
No	7 (23.3%)	5 (41.7%)	2 (11.1%)	
Yes	23 (76.7%)	7 (58.3%)	16 (88.9%)	0.084^b^
Partner status				
Unknown	42 (32.8%)	19 (32.8%)	23 (32.9%)	
Positive	86 (67.2%)	39 (67.2%)	47 (67.1%)	> 0.999
Disclosure of HIV status to partner				
No	10 (7.8%)	4 (6.9%)	6 (8.6%)	
Yes	118 (92.2%)	54 (93.1%)	64 (91.4%)	> 0.999^b^
Sex in exchange for compensation				
Never in the past month	115 (89.8%)	52 (89.7%)	63 (90.0%)	
Sometimes	12 (9.4%)	6 (10.3%)	6 (8.6%)	
All the time	1 (0.8%)	0 (0.0%)	1 (1.4%)	0.874^b^
Number of partners in the prior 3 months	1.00 (0.00)	1.00 (0.00)	1.00 (0.00)	0.608^a^
Condom use in the past month				
100% of the time	4 (4.3%)	1 (2.3%)	3 (5.9%)	
Less than 100% of the time	90 (95.7%)	42 (97.7%)	48 (94.1%)	0.395^b^
Time since diagnosis				
Less than a year ago	12 (9.4%)	5 (8.6%)	7 (10.0%)	
Between one and 5 years ago	75 (58.6%)	37 (63.8%)	38 (54.3%)	
Between 5 and 10 years ago	33 (25.8%)	14 (24.1%)	19 (27.1%)	
More than 10 years ago	8 (6.3%)	2 (3.4%)	6 (8.6%)	0.573^b^
CD4 cells per milliliter				
Less than 100	7 (5.5%)	2 (3.4%)	5 (7.1%)	
100 to 500	49 (38.3%)	23 (39.7%)	26 (37.1%)	
More than 500	50 (39.1%)	21 (36.2%)	29 (41.4%)	
I do not know	22 (17.2%)	12 (20.7%)	10 (14.3%)	0.623^a^

SOC+: Enhanced standard of care

Note. *USD* US dollars, *STI* sexually transmitted infection. ^a^Mann-Whitney U test was used. ^b^Fisher’s Exact test was used

Baseline self-reported use of IVP and laboratory parameters in the sample and by condition.

### Products used for IVP

Most women reported using water and soap for IVP (92% and 69% respectively), about half reported using a cloth, sponge or rag. About 20% of women used herbs or flowers from outdoors for IVP, and almost 30% used traditional medicines given by traditional healers for IVP. Women less often endorsed the use of lemon, vinegar or salt (Table 2).

**Table 2:** Intravaginal practices in the month prior to enrollment and laboratory parameters at baseline (vaginal flora, BV, HIV plasma and genital viral load, IL-6 and IL-8) by condition. (*n* = 128)

		Condition N(%) Median(IQR)		
	All	Experimental (*n* = 58)	SOC+ (*n* = 70)	*p*-value
Product use for IVP in the prior month				
Water				
No	10 (7.8%)	5 (8.6%)	5 (7.1%)	
Yes	118 (92.2%)	53 (91.4%)	65 (92.9%)	0.755^a^
Soap				
No	39 (30.5%)	22 (37.9%)	17 (24.3%)	
Yes	89 (69.5%)	36 (62.1%)	53 (75.7%)	0.095
Cloth, sponge or a rag				
No	33 (47.1%)	27 (46.6%)	33 (47.1%)	
Yes	68 (53.1%)	31 (53.4%)	37 (52.9%)	> 0.999
Traditional medicines				
No	91 (71.1%)	43 (74.1%)	48 (68.6%)	
Yes	37 (28.9%)	15 (25.9%)	22 (31.4%)	0.489
Herbs or flowers from outdoors				
No	103 (80.5%)	46 (79.3%)	57 (81.4%)	
Yes	25 (19.5%)	12 (20.7%)	13 (18.6%)	0.763
Lemon				
No	115 (89.3%)	52 (89.7%)	63 (90.0%)	
Yes	13 (10.2%)	6 (10.3%)	7 (10.0%)	> 0.999
Salt				
No	117 (91.4%)	54 (93.1%)	63 (90.0%)	
Yes	11 (8.6%)	4 (6.9%)	7 (10.0%)	0.753^a^
Vinegar				
No	124 (96.9%)	56 (96.6%)	68 (97.1%)	
Yes	4 (3.1%)	2 (3.4%)	2 (2.9%)	0.848^a^
Laboratory parameters				
Abnormal vaginal flora				
No	30 (23.4%)	16 (27.6%)	14 (20.0%)	
Yes	98 (76.6%)	42 (72.4%)	56 (80.0%)	0.213
Bacterial vaginosis				
No	47 (36.7%)	24 (41.4%)	23 (32.9%)	
Yes	81 (63.3%)	34 (58.6%)	47 (67.1%)	0.319
Plasma HIV RNA				
Undetectable	100 (78.1%)	46 (79.3%)	54 (77.1%)	
Detectable	28 (21.9%)	12 (20.7%)	16 (22.9%)	0.768
CVL HIV RNA				
Undetectable	108 (84.4%)	50 (86.2%)	58 (82.9%)	
Detectable	20 (15.6%)	8 (13.8%)	12 (17.1%)	0.603
Lower genital tract cytokines				
IL-6 (*n* = 68; Exp = 35, SOC+ = 33)	289.9 (258.0)	306.3 (313.7)	282.6 (249.1)	0.944
IL-8 (*n* = 68; Exp = 35, SOC+ = 33)	332.5 (1781.9)	380.11 (1311.9)	227.5 (2285.5)	**< 0.001**

CD4 cells/ml was self-reported

Plasma and CVL HIV RNA limit of detection was 20 copies/milliliter

*CVL* cervicovaginal lavage

*IVP* intravaginal practices

*SOC+* enhanced standard of care

Note. ^a^Fisher’s Exact test was used

### Laboratory parameters

At baseline, approximately three-fourths of the women sampled had abnormal vaginal flora (77%), nearly two-thirds (63%) had BV, and mean Nugent score was high (6.29). About one fifth of the women had detectable plasma HIV-1 VL, and only 15% had genital tract HIV-1 shedding. IVP, rates of abnormal vaginal flora, BV, plasma and genital HIV-1 RNA and cytokines did not differ between the two conditions except level of IL-8 which was higher in women enrolled in the SOC+ condition (Table 2).

### IVP and laboratory parameters

#### Self-reported IVP use over time and by condition

Results collapsed over condition showed that there was a reduction in the self-reported engagement of most IVPs, in particular in the use of water, soap, cloth, sponge or a rag from baseline to 6 months and 12 months. Self-reported use of soap, a cloth, sponge or rag increased from 6 months to 12 months. A reduction was seen in the self-reported use of herbs or flowers and traditional medicines from outdoors from baseline to 6 months, but this reduction was not sustained at 12 months. The changes in IVP over time among women enrolled in both conditions are illustrated in Table 3.

**Table 3:** Changes over time in the percentage of participants engaging in specific intravaginal practices and in laboratory parameters (vaginal flora, bacterial vaginosis, plasma, genital HIV RNA, IL-6, and IL-8). Plasma and CVL HIV RNA limit of detection was 20 copies/milliliter

	Baseline (*N* = 128) N(%) Median(IQR)	6-month (*N* = 90) N(%) Median(IQR)	12-month (*N* = 118) N(%) Median(IQR)	Baseline vs. 6-month *p*-value	Baseline vs. 12-month *p*-value	6-month vs. 12-month *p*-value
Products used for intravaginal practices in the prior month						
Water	91.7%	37.7%	47.2%	**< 0.001**	**< 0.001**	0.112
Soap	68.9%	11.8%	36.1%	**< 0.001**	**< 0.001**	**0.001**
Cloth, sponge or a rag	53.4%	6.3%	26.4%	**< 0.001**	**< 0.001**	**0.010**
Traditional medicine	28.5%	3.8%	30.6%	**0.001**	0.731	**< 0.001**
Herbs or flowers from outdoors	18.1%	5.0%	8.8%	**0.026**	0.070	0.363
Lemon	9.9%	2.2%	13.7%	0.050	**0.034**	**0.016**
Salt	8.7%	2.2%	6.6%	0.077	0.503	0.172
Vinegar	2.3%	2.2%	11.8%	0.962	**0.017**	**0.029**
Laboratory parameters						
Abnormal vaginal flora	77.3%	60.5%	58.2%	**0.014**	**0.003**	0.752
Bacterial vaginosis	64.2%	15.6%	23.6%	**< 0.001**	**< 0.001**	0.145
Detectable plasma HIV RNA	20.8%	14.9%	20.7%	0.147	0.995	0.374
Detectable CVL HIV RNA	13.8%	8.9%	8.9%	0.314	0.618	0.800
IL-6 (*n* = 68)	289.9 (258.0)	328.03 (264.8)	--	0.158	--	--
IL-8 (*n* = 68)	332.5 (1781.9)	311.4 (1400.3)	--	0.272	--	--

*CVL* cervicovaginal lavage

Bold: *p* < 0.05

Women in the experimental condition had non statistically significant reduction in the self-reported engagement in certain IVPs. When individual products were evaluated, women in the experimental condition had a greater reduction in the use of soap, cloth, sponge or rag and traditional medicines at 12 months, as illustrated in Table 4.

**Table 4:** Percentage of participants engaging in specific intravaginal practices and laboratory parameters (vaginal flora, bacterial vaginosis, plasma, genital HIV RNA, IL-6, and IL-8) by condition. Plasma and CVL HIV RNA limit of detection was 20 copies/milliliter

	Experimental (SOC+ plus group intervention)			Enhanced standard of care (SOC+)			SOC+ versus experimental	
	N(%) Median(IQR)			N(%) Median(IQR)				
	Baseline (*N* = 58)	6-month (*N* = 47)	12-month (*N* = 54)	Baseline (*N* = 70)	6-month (*N* = 53)	12-month (*N* = 64)	6-month *p*-value	12-month *p*-value
Products used for intravaginal practices in the prior month								
Water	90.9%	31.0%	40.7%	92.5%	45.8%	54.7%	0.159	0.140
Soap	63.6%	4.8%	27.8%	74.6%	29.2%	46.9%	**0.013**	**0.042**
Cloth, sponge or a rag	54.6%	2.4%	18.5%	52.2%	16.7%	37.5%	0.061	**0.031**
Traditional medicines	27.3%	2.4%	22.2%	29.9%	6.3%	42.2%	0.395	**0.029**
Herbs or flowers from outdoors	20.0%	2.4%	5.6%	16.4%	10.4%	14.1%	0.170	0.147
Lemon	10.9%	2.4%	9.3%	9.0%	2.1%	20.3%	0.924	0.111
Salt	7.3%	2.4%	5.6%	10.5%	2.1%	7.8%	0.924	0.629
Vinegar	3.6%	2.4%	7.4%	1.5%	2.1%	18.8%	0.924	0.090
Laboratory parameters								
Abnormal vaginal flora	72.7%	61.0%	66.7%	82.1%	60.0%	50.9%	0.926	0.090
Bacterial vaginosis	60.0%	22.0%	27.5%	68.7%	11.1%	20.3%	0.186	0.383
Detectable plasma HIV RNA	18.2%	11.9%	11.1%	23.9%	18.8%	38.9%	0.379	0.086
Detectable CVL HIV RNA	12.7%	5.0%	5.6%	14.9%	15.9%	20.0%	0.134	0.244
IL-6 (*n* = 68; Exp = 35, SOC+ = 33)	306.3 (313.7)	326.0 (313.1)	--	282.6 (249.1)	330.1 (261.6)	--	0.762	--
IL-8 (*n* = 68; Exp = 35, SOC+ = 33)	380.11 (1311.9)	328.0 (1423.5)	--	227.5 (2285.5)	254.2 (1385.5)	--	0.651	--

*SOC+* enhanced standard of care

*CVL* cervicovaginal lavage

Bold: *p* < 0.05

### Laboratory parameters over time and by condition

Results collapsed over condition showed that there was a decrease in the percentages of women with abnormal vaginal flora and with BV from baseline to 6 months and 12 months. No changes in plasma, CVL HIV-1 viral load, IL-6, and IL-8 were noted (see Table 3).

No changes were observed in rates of abnormal vaginal flora, BV, plasma, CVL HIV-1, viral load, IL-6, and IL-8 by condition (see Table 4).

For IL-6 and IL-8, we identified observations with extreme outliers. For IL-6, there was one outlier at 6-months. For IL-8, there were five: one at time baseline, and four at 6 month. However, because the results were identical with or without outliers, the results presented included the outliers to preserve the integrity and accuracy of the data and reported values.

## Discussion

This study compared two strategies to decrease intravaginal practices and bacterial vaginosis in HIV-infected women receiving antiretroviral therapy in urban Lusaka, Zambia. Both strategies provided diagnosis and treatment of BV, and a behavioral intervention to decrease IVP. The experimental condition differed from the enhanced standard of care in having an additional group intervention session. Results support the use of both strategies to enhance the uptake of recommendations to decrease IVP, and to reduce BV. Reductions in the self-reported use of soap, cloths, sponges and traditional medicines were greatest and maintained by women participating in the experimental condition. As seen in previous studies, the impact of the interventions decreased modestly over time, and some practices reverted back to baseline levels, but in general, benefits were maintained at 12 months post baseline.

Other interventions in sub-Saharan Africa have been effective in decreasing IVP, but this decreased was not accompanied by a decrease in BV [18, 19, 27, 28]. These studies have primarily addressed HIV-uninfected women who received intensive behavioral interventions with weekly visits to the study sites, and did not include treatment for BV. [17–19, 27]. Although screening asymptomatic women for BV has not been proven to be beneficial or feasible, we propose that prompt diagnosis and treatment for BV in women engaging in IVP appears to have an important effect on improving the vaginal flora, and may be necessary for the behavioral intervention to succeed in maintaining both healthy vaginal hygiene and flora. As we did not see a significant difference in BV in the experimental condition which included a group intervention when compared with enhanced standard of care (individual intervention), we proposed that an individual intervention easily administered by a health care provider can be incorporated into routine medical care of women with HIV who engage in IVP. Although most practices were decreased, the use of traditional medicines, lemon and vinegar was increased at follow up in the SOC+ condition, suggesting that some practices were replaced by others, and evaluation of interventions proposing alternative practices should be considered.

Our study is novel as it is focused on women with HIV infection, in whom the additional potential health consequences of IVP and BV include the risk of HIV transmission to sexual partners and newborns. In addition, study procedures included prompt diagnosis and treatment for BV, and assessment of genital HIV shedding and vaginal inflammation. Although no decrease in HIV genital shedding was observed after the intervention, it should be noted that all participants were receiving antiretrovirals, and it has previously been recognized that transmission in the setting of suppressed HIV viremia is unlikely to occur. [21, 29] However, viral loads are not regularly assessed in Zambia, and HIV-infected women may be unaware of their relative infectivity.

Additional research is merited to evaluate the effect of BV on the vaginal mucosa and the mechanism by which BV increases HIV transmission. It remains unclear whether asymptomatic screening or targeted intervention among HIV-infected women reporting use of IPV may be beneficial.

Some limitations must be considered in interpreting results from this study. Firstly, as a pilot study, the sample size was small, limiting the ability to detect differences in biological outcomes. We chose not to perform blocked randomization, which resulted in an allocation not close to 1:2 and may have resulted in some comparisons to be underpowered and we have closely adhered to the CONSORT guidelines for reporting [30]. Conducting the study in one city and in a single community health center study site limited the generalizability of the results. Women who were on hormonal contraception were excluded from this study, which further limited the generalizability of this study’s findings. Additionally, women in the experimental condition receiving HIV care at the facility may have discussed the group intervention with women enrolled in the SOC + condition, resulting in intrapractice contamination bias. Moreover, the lack of a true control arm hindered the assessment of a true intervention effect. Given the sexual nature of assessing IVP, it is possible that participants may have underreported IVP due to social-desirability. However, self-administered interviews (ACASI) were used to reduce the potential of reporting behaviors that may be perceived as socially desirable behavior. Lastly, as with other experimental designs, the results of this study may be affected due to pre and post comparisons and the Hawthorne or trial effects. Regarding biological outcomes, only BV and not other STIs were assessed, and are important co-factors for BV acquisition and may contribute to HIV genital shedding. The far apart spacing between visits may have missed episodes of BV and times where ART may have not been used.

In summary, we evaluated and compared strategies to decrease both IVP and BV in women with HIV infection. Notwithstanding the limitations of the findings, both strategies appeared effective, with possibly greater benefit obtained from the addition of a group intervention compared to a traditional one-on-one health information intervention.

## Conclusions

Interventions that could be easily administered during routine medical care and decrease IVP and BV are needed, and should be considered part of women’s health programs.

## Additional file

Additional file 1: Questionnaire: Intravaginal practices. (RTF 359 kb)

## Abbreviations

ACASI: Audio computer administered self-interviews
ART: Antiretroviral therapy
BV: Bacterial vaginosis
CIDRZ: Centre for Infectious Diseases Research in Zambia
CVL: Cervicovaginal lavage
GEE: Generalized estimating equations
IMB: Information motivation behavioral skills
IUD: Intrauterine device
IVP: Intravaginal practices
PCR: Polymerase chain reaction
RNA: Ribonucleic acid
SD: Standard deviation
SOC+: Enhanced standard of care.
STI: Sexually transmitted infection
