# Supplementary material for: A bio-behavioral intervention to decrease intravaginal practices and bacterial vaginosis among HIV infected Zambian women, a randomized pilot study
Source: BMC Infect Dis. 2017 May 12;17:338. doi: 10.1186/s12879-017-2436-3 (PMC5427559; doi:10.1186/s12879-017-2436-3)
Supplement: Additional file 1: — Questionnaire: Intravaginal practices. (RTF 359 kb) [file 12879_2017_2436_MOESM1_ESM.rtf]

A bio-behavioral intervention to decrease intravaginal practices and bacterial vaginosis among HIV infected Zambian women, a randomized pilot study
Alcaide, M.L., Chisembele, M., Malupande, E., Rodriguez, V.J., Fischl, M., Arheart, K., & Jones, D.L.
Q45.	When was the last time you put anything inside your vagina, such as your fingers, water, soap, a cloth, herbs, medicines, lemon, beer, salt, or made small cuts around the vagina?
VP4101	When was the last time you put anything inside your vagina, such as your fingers, water, soap, a cloth, herbs, medicines, lemon, beer, salt, or made small cuts around the vagina?	1
	1	=	In the last 2 days
	2	=	From 2 days to one week
	3	=	One-2 weeks ago
	4	=	two weeks to 1 month ago

Q46.	Why did you last put anything in your vagina, including your fingers, water, soap, a cloth, herbs, medicines, lemon, beer, or salt, or made small cuts around your vagina? 
Choose each that apply, you may choose more than one
VP4201A	Why did you last put anything in your vagina, including your fingers, water, soap, a cloth, herbs, medicines, lemon, beer, or salt, or made small cuts around your vagina?: To clean myself	1
	0	=	No
	1	=	Yes

VP4201B	Why did you last put anything in your vagina, including your fingers, water, soap, a cloth, herbs, medicines, lemon, beer, or salt, or made small cuts around your vagina?: To remove blood after menses	1
	0	=	No
	1	=	Yes

VP4201C	Why did you last put anything in your vagina, including your fingers, water, soap, a cloth, herbs, medicines, lemon, beer, or salt, or made small cuts around your vagina?: To remove sperm	1
	0	=	No
	1	=	Yes

VP4201D	Why did you last put anything in your vagina, including your fingers, water, soap, a cloth, herbs, medicines, lemon, beer, or salt, or made small cuts around your vagina?: To please my partner	1
	0	=	No
	1	=	Yes

VP4201E	Why did you last put anything in your vagina, including your fingers, water, soap, a cloth, herbs, medicines, lemon, beer, or salt, or made small cuts around your vagina?: To prevent pregnancy	1
	0	=	No
	1	=	Yes

VP4201F	Why did you last put anything in your vagina, including your fingers, water, soap, a cloth, herbs, medicines, lemon, beer, or salt, or made small cuts around your vagina?: To prevent or cure infections, including sexually transmitted infections	1
	0	=	No
	1	=	Yes

VP4201G	Why did you last put anything in your vagina, including your fingers, water, soap, a cloth, herbs, medicines, lemon, beer, or salt, or made small cuts around your vagina?: To prevent H I V.	1
	0	=	No
	1	=	Yes

VP4201H	Why did you last put anything in your vagina, including your fingers, water, soap, a cloth, herbs, medicines, lemon, beer, or salt, or made small cuts around your vagina?: I prefer not to answer	1
	0	=	No
	1	=	Yes


Q47.	What did you put inside your vagina in the past month? Choose all that apply, you may choose more than one
VP4301A	What did you put inside your vagina in the past month? Choose all that apply, you may choose more than one: My fingers	1
	0	=	No
	1	=	Yes

VP4301B	What did you put inside your vagina in the past month? Choose all that apply, you may choose more than one: Water	1
	0	=	No
	1	=	Yes

VP4301C	What did you put inside your vagina in the past month? Choose all that apply, you may choose more than one: Soap, lifeboy or other soap	1
	0	=	No
	1	=	Yes

VP4301D	What did you put inside your vagina in the past month? Choose all that apply, you may choose more than one: Cloth or sponge or paper or baby wipes	1
	0	=	No
	1	=	Yes

VP4301E	What did you put inside your vagina in the past month? Choose all that apply, you may choose more than one: Herbs or flowers from the garden	1
	0	=	No
	1	=	Yes

VP4301F	What did you put inside your vagina in the past month? Choose all that apply, you may choose more than one: Herbs or flowers, traditional medicines	1
	0	=	No
	1	=	Yes

VP4301G	What did you put inside your vagina in the past month? Choose all that apply, you may choose more than one: Lager Beer	1
	0	=	No
	1	=	Yes

VP4301H	What did you put inside your vagina in the past month? Choose all that apply, you may choose more than one: Ripe lemon	1
	0	=	No
	1	=	Yes

VP4301I	What did you put inside your vagina in the past month? Choose all that apply, you may choose more than one: Vinegar	1
	0	=	No
	1	=	Yes

VP4301J	What did you put inside your vagina in the past month? Choose all that apply, you may choose more than one: Salt	1
	0	=	No
	1	=	Yes

VP4301K	What did you put inside your vagina in the past month? Choose all that apply, you may choose more than one: I made small cuts around the vagina	1
	0	=	No
	1	=	Yes

VP4301L	What did you put inside your vagina in the past month? Choose all that apply, you may choose more than one: I prefer not to answer	1
	0	=	No
	1	=	Yes


Q48.	How likely would you be to engage in Vaginal practices if they: Are BAD for your HYGIENE, Your PARTNER does NOT LIKE them, Have NO EFFECT on your HEALTH
VP4401	How likely would you be to engage in Vaginal practices if they: Are BAD for your HYGIENE, Your PARTNER does NOT LIKE them, Have NO EFFECT on your HEALTH	2
	1	=	Very unlikely
	2 - 8	=	unlabelled scale points
	9	=	Very likely

Q49.	How likely would you be to engage in Vaginal practices if they: Are GOOD for your HYGIENE, Your PARTNER LIKES them, Have NO EFFECT on your HEALTH
VP4501	How likely would you be to engage in Vaginal practices if they: Are GOOD for your HYGIENE, Your PARTNER LIKES them, Have NO EFFECT on your HEALTH	2
	1	=	Very unlikely
	2 - 8	=	unlabelled scale points
	9	=	Very likely

Q50.	How likely would you be to engage in Vaginal practices if they: Have NO EFFECT on your HYGIENE, Your PARTNER DOES NOT CARE, Have NO EFFECT on your HEALTH
VP4601	How likely would you be to engage in Vaginal practices if they: Have NO EFFECT on your HYGIENE, Your PARTNER DOES NOT CARE, Have NO EFFECT on your HEALTH	2
	1	=	Very unlikely
	2 - 8	=	unlabelled scale points
	9	=	Very likely

Q51.	How old were you when you were taught about vaginal practices? age in years. If you don't remember exactly, please guess.
VP4701	How old were you when you were taught about vaginal practices? age in years. If you don't remember exactly, please guess.	2
	0 - 96	=	range

Q52.	How old were you the first time you put something inside your vagina? Age in years. If you don't remember exactly, please guess.
VP4801	How old were you the first time you put something inside your vagina? Age in years. If you don't remember exactly, please guess.	2
	0 - 96	=	range

Q53.	Did you do vaginal practices to clean yourself?
VP49A101	Did you do vaginal practices to clean yourself?	1
	0	=	No
	1	=	Yes

Q54.	At what age did you first do vaginal practices to clean yourself: age in years?
VP49A201	At what age did you first do vaginal practices to clean yourself: age in years?	2
	0 - 96	=	range

Q55.	Did you do vaginal practices to remove blood after period or menses?
VP49B101	Did you do vaginal practices to remove blood after period or menses?	1
	0	=	No
	1	=	Yes

Q56.	At what age did you first do vaginal practices to remove blood after period or menses? age in years.
VP49B201	At what age did you first do vaginal practices to remove blood after period or menses? age in years	2
	0 - 96	=	range

Q57.	Did you do vaginal practices to please your sexual partner, husband or boyfriend?
VP49C101	Did you do vaginal practices to please your sexual partner, husband or boyfriend?	1
	0	=	No
	1	=	Yes

Q58.	At what age did you first do vaginal practices to please your sexual partner, husband or boyfriend? age in years.
VP49C201	At what age did you first do vaginal practices to please your sexual partner, husband or boyfriend? age in years.	2
	0 - 96	=	range

Q59.	Did you do vaginal practices to prevent infections?
VP49D101	Did you do vaginal practices to prevent infections?	1
	0	=	No
	1	=	Yes

Q60.	At what age did you first do vaginal practices to prevent infections? age in years.
VP49D201	At what age did you first do vaginal practices to prevent infections? age in years.	2
	0 - 96	=	range

Q61.	Did you do vaginal practices to prevent pregnancy?
VP49E101	Did you do vaginal practices to prevent pregnancy?	1
	0	=	No
	1	=	Yes

Q62.	At what age did you first do vaginal practices to prevent pregnancy? Age in years.
VP49E201	At what age did you first do vaginal practices to prevent pregnancy? Age in years.	2
	0 - 96	=	range

Q63.	Who was the person that told you about these vaginal practices? Choose all that apply, you may choose more than one
VP5001A	Who was the person that told you about these vaginal practices? Choose all that apply, you may choose more than one: Mother	1
	0	=	No
	1	=	Yes

VP5001B	Who was the person that told you about these vaginal practices? Choose all that apply, you may choose more than one: Relative - aunty	1
	0	=	No
	1	=	Yes

VP5001C	Who was the person that told you about these vaginal practices? Choose all that apply, you may choose more than one: Friend	1
	0	=	No
	1	=	Yes

VP5001D	Who was the person that told you about these vaginal practices? Choose all that apply, you may choose more than one: Alangizi	1
	0	=	No
	1	=	Yes

VP5001E	Who was the person that told you about these vaginal practices? Choose all that apply, you may choose more than one: Doctor or nurse	1
	0	=	No
	1	=	Yes

VP5001F	Who was the person that told you about these vaginal practices? Choose all that apply, you may choose more than one: It was my own idea	1
	0	=	No
	1	=	Yes


Q64.	How likely would you be to engage in Vaginal practices if they: Are GOOD for your HYGIENE, Your PARTNER DOES NOT CARE, Are BAD for your HEALTH
VP5101	How likely would you be to engage in Vaginal practices if they: Are GOOD for your HYGIENE, Your PARTNER DOES NOT CARE, Are BAD for your HEALTH	2
	1	=	Very unlikely
	2 - 8	=	unlabelled scale points
	9	=	Very likely

Q65.	How likely would you be to engage in Vaginal practices if they: Have NO EFFECT on your HYGIENE, Your PARTNER LIKES , Are GOOD for your HEALTH
VP5201	How likely would you be to engage in Vaginal practices if they: Have NO EFFECT on your HYGIENE, Your PARTNER LIKES , Are GOOD for your HEALTH	2
	1	=	Very unlikely
	2 - 8	=	unlabelled scale points
	9	=	Very likely

Q66.	How likely would you be to engage in Vaginal practices if they: Are BAD for your HYGIENE, Your PARTNER DOES NOT CARE, Are GOOD for your HEALTH
VP5301	How likely would you be to engage in Vaginal practices if they: Are BAD for your HYGIENE, Your PARTNER DOES NOT CARE, Are GOOD for your HEALTH	2
	1	=	Very unlikely
	2 - 8	=	unlabelled scale points
	9	=	Very likely

Q67.	Have you put your fingers inside your vagina in the last month
VP54A01	Have you put your fingers inside your vagina in the last month	1
	0	=	No
	1	=	Yes
	2	=	I prefer not to answer

Q68.	When was the last time you put your fingers in the vagina?
VP54B01	When was the last time you put your fingers in the vagina?	1
	1	=	within the last 2 days
	2	=	from 2 days to one week
	3	=	one week to 2 weeks
	4	=	two weeks to 1 month
	5	=	I prefer not to answer

Q69.	How often do you put your fingers in your vagina?
VP54C01	How often do you put your fingers in your vagina?	1
	1	=	daily
	2	=	weekly
	3	=	approximately once a month
	4	=	I prefer not to answer

Q70.	Why did you insert your fingers in your vagina? Choose all that apply, you may choose more than one
VP54D01A	Why did you insert your fingers in your vagina? Choose all that apply, you may choose more than one: to clean myself	1
	0	=	No
	1	=	Yes

VP54D01B	Why did you insert your fingers in your vagina? Choose all that apply, you may choose more than one: to get rid of a discharge, to clean away a fluid	1
	0	=	No
	1	=	Yes

VP54D01C	Why did you insert your fingers in your vagina? Choose all that apply, you may choose more than one: to get rid of an odor	1
	0	=	No
	1	=	Yes

VP54D01D	Why did you insert your fingers in your vagina? Choose all that apply, you may choose more than one: to clean blood after menses, to remove blood	1
	0	=	No
	1	=	Yes

VP54D01E	Why did you insert your fingers in your vagina? Choose all that apply, you may choose more than one: to prevent pregnancy	1
	0	=	No
	1	=	Yes

VP54D01F	Why did you insert your fingers in your vagina? Choose all that apply, you may choose more than one: to prevent a sexually transmitted infection, an S T I	1
	0	=	No
	1	=	Yes

VP54D01G	Why did you insert your fingers in your vagina? Choose all that apply, you may choose more than one: to prevent H I V	1
	0	=	No
	1	=	Yes

VP54D01H	Why did you insert your fingers in your vagina? Choose all that apply, you may choose more than one: to please my partner, husband or boyfriend	1
	0	=	No
	1	=	Yes

VP54D01I	Why did you insert your fingers in your vagina? Choose all that apply, you may choose more than one: I prefer not to answer	1
	0	=	No
	1	=	Yes


Q71.	Have you put water inside your vagina in the last month?
VP55A01	Have you put water inside your vagina in the last month?	1
	0	=	No
	1	=	Yes
	2	=	I prefer not to answer

Q72.	When was the last time you put water in your vagina?
VP55B01	When was the last time you put water in your vagina?	1
	1	=	within the last 2 days
	2	=	from 2 days to one week
	3	=	one week to 2 weeks
	4	=	two weeks to 1 month
	5	=	I prefer not to answer

Q73.	How often do you put water in your vagina?
VP55C01	How often do you put water in your vagina?	1
	1	=	daily
	2	=	weekly
	3	=	approximately once a month
	4	=	I prefer not to answer

Q74.	Why did you put water inside your vagina? Choose all that apply, you may choose more than one
VP55D01A	Why did you put water inside your vagina? Choose all that apply, you may choose more than one: to clean myself	1
	0	=	No
	1	=	Yes

VP55D01B	Why did you put water inside your vagina? Choose all that apply, you may choose more than one: to get rid of a discharge, to clean away a fluid	1
	0	=	No
	1	=	Yes

VP55D01C	Why did you put water inside your vagina? Choose all that apply, you may choose more than one: to get rid of an odor	1
	0	=	No
	1	=	Yes

VP55D01D	Why did you put water inside your vagina? Choose all that apply, you may choose more than one: to clean blood after menses, to remove blood	1
	0	=	No
	1	=	Yes

VP55D01E	Why did you put water inside your vagina? Choose all that apply, you may choose more than one: to prevent pregnancy	1
	0	=	No
	1	=	Yes

VP55D01F	Why did you put water inside your vagina? Choose all that apply, you may choose more than one: to prevent a sexually transmitted infection, an S T I	1
	0	=	No
	1	=	Yes

VP55D01G	Why did you put water inside your vagina? Choose all that apply, you may choose more than one: to prevent H I V	1
	0	=	No
	1	=	Yes

VP55D01H	Why did you put water inside your vagina? Choose all that apply, you may choose more than one: to please my partner, husband or boyfriend	1
	0	=	No
	1	=	Yes

VP55D01I	Why did you put water inside your vagina? Choose all that apply, you may choose more than one: I prefer not to answer	1
	0	=	No
	1	=	Yes


Q75.	Have you put soap inside your vagina in the last month?
VP56A01	Have you put soap inside your vagina in the last month?	1
	0	=	No
	1	=	Yes
	2	=	I prefer not to answer

Q76.	When was the last time you put soap in your vagina?
VP56B01	When was the last time you put soap in your vagina?	1
	1	=	within the last 2 days
	2	=	from 2 days to one week
	3	=	one week to 2 weeks
	4	=	two weeks to 1 month
	5	=	I prefer not to answer

Q77.	How often do you put soap in your vagina?
VP56C01	How often do you put soap in your vagina?	1
	1	=	daily
	2	=	weekly
	3	=	approximately once a month
	4	=	I prefer not to answer

Q78.	Why did you put soap inside your vagina? Choose all that apply, you may choose more   than one
VP56D01A	Why did you put soap inside your vagina? Choose all that apply, you may choose more   than one: to clean myself	1
	0	=	No
	1	=	Yes

VP56D01B	Why did you put soap inside your vagina? Choose all that apply, you may choose more   than one: to get rid of a discharge, to clean away a fluid	1
	0	=	No
	1	=	Yes

VP56D01C	Why did you put soap inside your vagina? Choose all that apply, you may choose more   than one: to get rid of an odor	1
	0	=	No
	1	=	Yes

VP56D01D	Why did you put soap inside your vagina? Choose all that apply, you may choose more   than one: to clean blood after menses, to remove blood	1
	0	=	No
	1	=	Yes

VP56D01E	Why did you put soap inside your vagina? Choose all that apply, you may choose more   than one: to prevent pregnancy	1
	0	=	No
	1	=	Yes

VP56D01F	Why did you put soap inside your vagina? Choose all that apply, you may choose more   than one: to prevent a sexually transmitted infection, an S T I	1
	0	=	No
	1	=	Yes

VP56D01G	Why did you put soap inside your vagina? Choose all that apply, you may choose more   than one: to prevent H I V	1
	0	=	No
	1	=	Yes

VP56D01H	Why did you put soap inside your vagina? Choose all that apply, you may choose more   than one: to please my partner, husband or boyfriend	1
	0	=	No
	1	=	Yes

VP56D01I	Why did you put soap inside your vagina? Choose all that apply, you may choose more   than one: I prefer not to answer	1
	0	=	No
	1	=	Yes


Q79.	Have you put cloth, paper, wipes or sponge inside your vagina in the last month?
VP57A01	Have you put cloth, paper, wipes or sponge inside your vagina in the last month?	1
	0	=	No
	1	=	Yes
	2	=	I prefer not to answer

Q80.	When was the last time you put cloth, paper, wipes or sponge in your vagina?
VP57B01	When was the last time you put cloth, paper, wipes or sponge in your vagina?	1
	1	=	within the last 2 days
	2	=	from 2 days to one week
	3	=	one week to 2 weeks
	4	=	two weeks to 1 month
	5	=	I prefer not to answer

Q81.	How often did you put cloth, paper, wipes or sponge in your vagina?
VP57C01	How often did you put cloth, paper, wipes or sponge in your vagina?	1
	1	=	daily
	2	=	weekly
	3	=	approximately once a month
	4	=	I prefer not to answer

Q82.	Why did you put cloth, paper, wipes or sponge inside your vagina? Choose all that apply, you may choose more than one
VP57D01A	Why did you put cloth, paper, wipes or sponge inside your vagina? Choose all that apply, you may choose more than one: to clean myself	1
	0	=	No
	1	=	Yes

VP57D01B	Why did you put cloth, paper, wipes or sponge inside your vagina? Choose all that apply, you may choose more than one: to get rid of a discharge, to clean away a fluid	1
	0	=	No
	1	=	Yes

VP57D01C	Why did you put cloth, paper, wipes or sponge inside your vagina? Choose all that apply, you may choose more than one: to get rid of an odor	1
	0	=	No
	1	=	Yes

VP57D01D	Why did you put cloth, paper, wipes or sponge inside your vagina? Choose all that apply, you may choose more than one: to clean blood after menses, to remove blood	1
	0	=	No
	1	=	Yes

VP57D01E	Why did you put cloth, paper, wipes or sponge inside your vagina? Choose all that apply, you may choose more than one: to prevent pregnancy	1
	0	=	No
	1	=	Yes

VP57D01F	Why did you put cloth, paper, wipes or sponge inside your vagina? Choose all that apply, you may choose more than one: to prevent a sexually transmitted infection, an S T I	1
	0	=	No
	1	=	Yes

VP57D01G	Why did you put cloth, paper, wipes or sponge inside your vagina? Choose all that apply, you may choose more than one: to prevent H I V	1
	0	=	No
	1	=	Yes

VP57D01H	Why did you put cloth, paper, wipes or sponge inside your vagina? Choose all that apply, you may choose more than one: to please my partner, husband or boyfriend	1
	0	=	No
	1	=	Yes

VP57D01I	Why did you put cloth, paper, wipes or sponge inside your vagina? Choose all that apply, you may choose more than one: I prefer not to answer	1
	0	=	No
	1	=	Yes


Q83.	Have you put herbs of flowers from the garden inside your vagina in the last month?
VP58A01	Have you put herbs of flowers from the garden inside your vagina in the last month?	1
	0	=	No
	1	=	Yes
	2	=	I prefer not to answer

Q84.	When was the last time you put herbs of flowers from the garden in your vagina?
VP58B01	When was the last time you put herbs of flowers from the garden in your vagina?	1
	1	=	within the last 2 days
	2	=	from 2 days to one week
	3	=	one week to 2 weeks
	4	=	two weeks to 1 month
	5	=	I prefer not to answer

Q85.	How often did you put herbs of flowers from the garden in your vagina?
VP58C01	How often did you put herbs of flowers from the garden in your vagina?	1
	1	=	daily
	2	=	weekly
	3	=	approximately once a month
	4	=	I prefer not to answer

Q86.	Why did you put herbs of flowers from the garden inside your Choose all that apply, you may choose more than one
VP58D01A	Why did you put herbs of flowers from the garden inside your Choose all that apply, you may choose more than one: to clean myself	1
	0	=	No
	1	=	Yes

VP58D01B	Why did you put herbs of flowers from the garden inside your Choose all that apply, you may choose more than one: to get rid of a discharge, to clean away a fluid	1
	0	=	No
	1	=	Yes

VP58D01C	Why did you put herbs of flowers from the garden inside your Choose all that apply, you may choose more than one: to get rid of an odor	1
	0	=	No
	1	=	Yes

VP58D01D	Why did you put herbs of flowers from the garden inside your Choose all that apply, you may choose more than one: to clean blood after menses, to remove blood	1
	0	=	No
	1	=	Yes

VP58D01E	Why did you put herbs of flowers from the garden inside your Choose all that apply, you may choose more than one: to prevent pregnancy	1
	0	=	No
	1	=	Yes

VP58D01F	Why did you put herbs of flowers from the garden inside your Choose all that apply, you may choose more than one: to prevent a sexually transmitted infection, an S T I	1
	0	=	No
	1	=	Yes

VP58D01G	Why did you put herbs of flowers from the garden inside your Choose all that apply, you may choose more than one: to prevent H I V	1
	0	=	No
	1	=	Yes

VP58D01H	Why did you put herbs of flowers from the garden inside your Choose all that apply, you may choose more than one: to please my partner, husband or boyfriend	1
	0	=	No
	1	=	Yes

VP58D01I	Why did you put herbs of flowers from the garden inside your Choose all that apply, you may choose more than one: I prefer not to answer	1
	0	=	No
	1	=	Yes


Q87.	Have you put herbs that are traditional medicines inside your vagina in the last month?
VP59A01	Have you put herbs that are traditional medicines inside your vagina in the last month?	1
	0	=	No
	1	=	Yes
	2	=	I prefer not to answer

Q88.	When was the last time you put herbs that are traditional medicines in your vagina?
VP59B01	When was the last time you put herbs that are traditional medicines in your vagina?	1
	1	=	within the last 2 days
	2	=	from 2 days to one week
	3	=	one week to 2 weeks
	4	=	two weeks to 1 month
	5	=	I prefer not to answer

Q89.	How often did you put herbs that are traditional medicines in your vagina?
VP59C01	How often did you put herbs that are traditional medicines in your vagina?	1
	1	=	daily
	2	=	weekly
	3	=	approximately once a month
	4	=	I prefer not to answer

Q90.	Why did you put herbs that are traditional medicines inside your vagina  Choose all that apply, you may choose more than one
VP59D01A	Why did you put herbs that are traditional medicines inside your vagina  Choose all that apply, you may choose more than one: to clean myself	1
	0	=	No
	1	=	Yes

VP59D01B	Why did you put herbs that are traditional medicines inside your vagina  Choose all that apply, you may choose more than one: to get rid of a discharge, to clean away a fluid	1
	0	=	No
	1	=	Yes

VP59D01C	Why did you put herbs that are traditional medicines inside your vagina  Choose all that apply, you may choose more than one: to get rid of an odor	1
	0	=	No
	1	=	Yes

VP59D01D	Why did you put herbs that are traditional medicines inside your vagina  Choose all that apply, you may choose more than one: to clean blood after menses, to remove blood	1
	0	=	No
	1	=	Yes

VP59D01E	Why did you put herbs that are traditional medicines inside your vagina  Choose all that apply, you may choose more than one: to prevent pregnancy	1
	0	=	No
	1	=	Yes

VP59D01F	Why did you put herbs that are traditional medicines inside your vagina  Choose all that apply, you may choose more than one: to prevent a sexually transmitted infection, an S T I	1
	0	=	No
	1	=	Yes

VP59D01G	Why did you put herbs that are traditional medicines inside your vagina  Choose all that apply, you may choose more than one: to prevent H I V	1
	0	=	No
	1	=	Yes

VP59D01H	Why did you put herbs that are traditional medicines inside your vagina  Choose all that apply, you may choose more than one: to please my partner, husband or boyfriend	1
	0	=	No
	1	=	Yes

VP59D01I	Why did you put herbs that are traditional medicines inside your vagina  Choose all that apply, you may choose more than one: I prefer not to answer	1
	0	=	No
	1	=	Yes


Q91.	Have you put lager beer inside your vagina in the last month?
VP60A01	Have you put lager beer inside your vagina in the last month?	1
	0	=	No
	1	=	Yes
	2	=	I prefer not to answer

Q92.	When was the last time you put lager beer in your vagina?
VP60B01	When was the last time you put lager beer in your vagina?	1
	1	=	within the last 2 days
	2	=	from 2 days to one week
	3	=	one week to 2 weeks
	4	=	two weeks to 1 month
	5	=	I prefer not to answer

Q93.	How often do you put lager beer in your vagina?
VP60C01	How often do you put lager beer in your vagina?	1
	1	=	daily
	2	=	weekly
	3	=	approximately once a month
	4	=	I prefer not to answer

Q94.	Why did you put lager beer inside your vagina? Choose all that apply, you may choose more than one
VP60D01A	Why did you put lager beer inside your vagina? Choose all that apply, you may choose more than one: to clean myself	1
	0	=	No
	1	=	Yes

VP60D01B	Why did you put lager beer inside your vagina? Choose all that apply, you may choose more than one: to get rid of a discharge, to clean away a fluid	1
	0	=	No
	1	=	Yes

VP60D01C	Why did you put lager beer inside your vagina? Choose all that apply, you may choose more than one: to get rid of an odor	1
	0	=	No
	1	=	Yes

VP60D01D	Why did you put lager beer inside your vagina? Choose all that apply, you may choose more than one: to clean blood after menses, to remove blood	1
	0	=	No
	1	=	Yes

VP60D01E	Why did you put lager beer inside your vagina? Choose all that apply, you may choose more than one: to prevent pregnancy	1
	0	=	No
	1	=	Yes

VP60D01F	Why did you put lager beer inside your vagina? Choose all that apply, you may choose more than one: to prevent a sexually transmitted infection, an S T I	1
	0	=	No
	1	=	Yes

VP60D01G	Why did you put lager beer inside your vagina? Choose all that apply, you may choose more than one: to prevent H I V	1
	0	=	No
	1	=	Yes

VP60D01H	Why did you put lager beer inside your vagina? Choose all that apply, you may choose more than one: to please my partner, husband or boyfriend	1
	0	=	No
	1	=	Yes

VP60D01I	Why did you put lager beer inside your vagina? Choose all that apply, you may choose more than one: I prefer not to answer	1
	0	=	No
	1	=	Yes


Q95.	Have you put ripe lemon inside your vagina in the last month?
VP61A01	Have you put ripe lemon inside your vagina in the last month?	1
	0	=	No
	1	=	Yes
	2	=	I prefer not to answer

Q96.	When was the last time you put ripe lemon in your vagina?
VP61B01	When was the last time you put ripe lemon in your vagina?	1
	1	=	within the last 2 days
	2	=	from 2 days to one week
	3	=	one week to 2 weeks
	4	=	two weeks to 1 month
	5	=	I prefer not to answer

Q97.	How often do you put ripe lemon in your vagina?
VP61C01	How often do you put ripe lemon in your vagina?	1
	1	=	daily
	2	=	weekly
	3	=	approximately once a month
	4	=	I prefer not to answer

Q98.	Why did you put ripe lemon inside your vagina in the past month? Choose all that apply, you may choose more than one
VP61D01A	Why did you put ripe lemon inside your vagina in the past month? Choose all that apply, you may choose more than one: to clean myself	1
	0	=	No
	1	=	Yes

VP61D01B	Why did you put ripe lemon inside your vagina in the past month? Choose all that apply, you may choose more than one: to get rid of a discharge, to clean away a fluid	1
	0	=	No
	1	=	Yes

VP61D01C	Why did you put ripe lemon inside your vagina in the past month? Choose all that apply, you may choose more than one: to get rid of an odor	1
	0	=	No
	1	=	Yes

VP61D01D	Why did you put ripe lemon inside your vagina in the past month? Choose all that apply, you may choose more than one: to clean blood after menses, to remove blood	1
	0	=	No
	1	=	Yes

VP61D01E	Why did you put ripe lemon inside your vagina in the past month? Choose all that apply, you may choose more than one: to prevent pregnancy	1
	0	=	No
	1	=	Yes

VP61D01F	Why did you put ripe lemon inside your vagina in the past month? Choose all that apply, you may choose more than one: to prevent a sexually transmitted infection, an S T I	1
	0	=	No
	1	=	Yes

VP61D01G	Why did you put ripe lemon inside your vagina in the past month? Choose all that apply, you may choose more than one: to prevent H I V	1
	0	=	No
	1	=	Yes

VP61D01H	Why did you put ripe lemon inside your vagina in the past month? Choose all that apply, you may choose more than one: to please my partner, husband or boyfriend	1
	0	=	No
	1	=	Yes

VP61D01I	Why did you put ripe lemon inside your vagina in the past month? Choose all that apply, you may choose more than one: I prefer not to answer	1
	0	=	No
	1	=	Yes


Q99.	Have you put vinegar inside your vagina in the last month?
VP62A01	Have you put vinegar inside your vagina in the last month?	1
	0	=	No
	1	=	Yes
	2	=	I prefer not to answer

Q100.	When was the last time you put vinegar in your vagina?
VP62B01	When was the last time you put vinegar in your vagina?	1
	1	=	within the last 2 days
	2	=	from 2 days to one week
	3	=	one week to 2 weeks
	4	=	two weeks to 1 month
	5	=	I prefer not to answer

Q101.	How often do you put vinegar in your vagina?
VP62C01	How often do you put vinegar in your vagina?	1
	1	=	daily
	2	=	weekly
	3	=	approximately once a month
	4	=	I prefer not to answer

Q102.	Why did you put vinegar inside your vagina? Choose all that apply, you may choose more than one
VP62D01A	Why did you put vinegar inside your vagina? Choose all that apply, you may choose more than one: to clean myself	1
	0	=	No
	1	=	Yes

VP62D01B	Why did you put vinegar inside your vagina? Choose all that apply, you may choose more than one: to get rid of a discharge, to clean away a fluid	1
	0	=	No
	1	=	Yes

VP62D01C	Why did you put vinegar inside your vagina? Choose all that apply, you may choose more than one: to get rid of an odor	1
	0	=	No
	1	=	Yes

VP62D01D	Why did you put vinegar inside your vagina? Choose all that apply, you may choose more than one: to clean blood after menses, to remove blood	1
	0	=	No
	1	=	Yes

VP62D01E	Why did you put vinegar inside your vagina? Choose all that apply, you may choose more than one: to prevent pregnancy	1
	0	=	No
	1	=	Yes

VP62D01F	Why did you put vinegar inside your vagina? Choose all that apply, you may choose more than one: to prevent a sexually transmitted infection, an S T I	1
	0	=	No
	1	=	Yes

VP62D01G	Why did you put vinegar inside your vagina? Choose all that apply, you may choose more than one: to prevent H I V	1
	0	=	No
	1	=	Yes

VP62D01H	Why did you put vinegar inside your vagina? Choose all that apply, you may choose more than one: to please my partner, husband or boyfriend	1
	0	=	No
	1	=	Yes

VP62D01I	Why did you put vinegar inside your vagina? Choose all that apply, you may choose more than one: I prefer not to answer	1
	0	=	No
	1	=	Yes


Q103.	Have you put salt inside your vagina in the last month?
VP63A01	Have you put salt inside your vagina in the last month?	1
	0	=	No
	1	=	Yes
	2	=	I prefer not to answer

Q104.	When was the last time you put salt in your vagina?
VP63B01	When was the last time you put salt in your vagina?	1
	1	=	within the last 2 days
	2	=	from 2 days to one week
	3	=	one week to 2 weeks
	4	=	two weeks to 1 month
	5	=	I prefer not to answer

Q105.	How often did you put salt in your vagina?
VP63C01	How often did you put salt in your vagina?	1
	1	=	daily
	2	=	weekly
	3	=	approximately once a month
	4	=	I prefer not to answer

Q106.	Why did you put salt inside your vagina in the past month? Choose all that apply, you may choose more than one
VP63D01A	Why did you put salt inside your vagina in the past month? Choose all that apply, you may choose more than one: to clean myself	1
	0	=	No
	1	=	Yes

VP63D01B	Why did you put salt inside your vagina in the past month? Choose all that apply, you may choose more than one: to get rid of a discharge, to clean away a fluid	1
	0	=	No
	1	=	Yes

VP63D01C	Why did you put salt inside your vagina in the past month? Choose all that apply, you may choose more than one: to get rid of an odor	1
	0	=	No
	1	=	Yes

VP63D01D	Why did you put salt inside your vagina in the past month? Choose all that apply, you may choose more than one: to clean blood after menses, to remove blood	1
	0	=	No
	1	=	Yes

VP63D01E	Why did you put salt inside your vagina in the past month? Choose all that apply, you may choose more than one: to prevent pregnancy	1
	0	=	No
	1	=	Yes

VP63D01F	Why did you put salt inside your vagina in the past month? Choose all that apply, you may choose more than one: to prevent a sexually transmitted infection, an S T I	1
	0	=	No
	1	=	Yes

VP63D01G	Why did you put salt inside your vagina in the past month? Choose all that apply, you may choose more than one: to prevent H I V	1
	0	=	No
	1	=	Yes

VP63D01H	Why did you put salt inside your vagina in the past month? Choose all that apply, you may choose more than one: to please my partner, husband or boyfriend	1
	0	=	No
	1	=	Yes

VP63D01I	Why did you put salt inside your vagina in the past month? Choose all that apply, you may choose more than one: I prefer not to answer	1
	0	=	No
	1	=	Yes


Q107.	Have you done any small cuts around the vagina in the last month?
VP64A01	Have you done any small cuts around the vagina in the last month?	1
	0	=	No
	1	=	Yes
	2	=	I prefer not to answer

Q108.	When was the last time you did small cuts around vagina?
VP64B01	When was the last time you did small cuts around vagina?	1
	1	=	within the last 2 days
	2	=	from 2 days to one week
	3	=	one week to 2 weeks
	4	=	two weeks to 1 month
	5	=	I prefer not to answer

Q109.	How often do you do small cuts around  the vagina?
VP64C01	How often do you do small cuts around  the vagina?	1
	1	=	daily
	2	=	weekly
	3	=	approximately once a month
	4	=	I prefer not to answer

Q110.	Why have done small cuts around the vagina in the past month? Choose all that apply, you may choose more than one
VP64D01A	Why have done small cuts around the vagina in the past month? Choose all that apply, you may choose more than one: to clean myself	1
	0	=	No
	1	=	Yes

VP64D01B	Why have done small cuts around the vagina in the past month? Choose all that apply, you may choose more than one: to get rid of a discharge, to clean away a fluid	1
	0	=	No
	1	=	Yes

VP64D01C	Why have done small cuts around the vagina in the past month? Choose all that apply, you may choose more than one: to get rid of an odor	1
	0	=	No
	1	=	Yes

VP64D01D	Why have done small cuts around the vagina in the past month? Choose all that apply, you may choose more than one: to clean blood after menses, to remove blood	1
	0	=	No
	1	=	Yes

VP64D01E	Why have done small cuts around the vagina in the past month? Choose all that apply, you may choose more than one: to prevent pregnancy	1
	0	=	No
	1	=	Yes

VP64D01F	Why have done small cuts around the vagina in the past month? Choose all that apply, you may choose more than one: to prevent a sexually transmitted infection, an S T I	1
	0	=	No
	1	=	Yes

VP64D01G	Why have done small cuts around the vagina in the past month? Choose all that apply, you may choose more than one: to prevent H I V	1
	0	=	No
	1	=	Yes

VP64D01H	Why have done small cuts around the vagina in the past month? Choose all that apply, you may choose more than one: to please my partner, husband or boyfriend	1
	0	=	No
	1	=	Yes

VP64D01I	Why have done small cuts around the vagina in the past month? Choose all that apply, you may choose more than one: I prefer not to answer	1
	0	=	No
	1	=	Yes


Q111.	How likely would you be to engage in Vaginal practices if they: Are BAD for your HYGIENE, Your PARTNER LIKES, Are BAD for your HEALTH
VP6501	How likely would you be to engage in Vaginal practices if they: Are BAD for your HYGIENE, Your PARTNER LIKES, Are BAD for your HEALTH	2
	1	=	very unlikely
	2 - 8	=	unlabelled scale points
	9	=	Very likely

Q112.	How likely would you be to engage in Vaginal practices if they: Are GOOD for your HYGIENE, Your PARTNER DOES not like, Are GOOD for your HEALTH
VP6601	How likely would you be to engage in Vaginal practices if they: Are GOOD for your HYGIENE, Your PARTNER DOES not like, Are GOOD for your HEALTH	2
	1	=	Very unlikely
	2 - 8	=	unlabelled scale points
	9	=	Very likely

Q113.	How likely would you be to engage in Vaginal practices if they: Have NO EFFECT on your HYGIENE, Your PARTNERDOES NOT like, Are BAD for your HEALTH
VP6701	How likely would you be to engage in Vaginal practices if they: Have NO EFFECT on your HYGIENE, Your PARTNERDOES NOT like, Are BAD for your HEALTH	2
	1	=	Very unlikely
	2 - 8	=	unlabelled scale points
	9	=	Very likely

Q114.	What do you think it might happen if you stop the vaginal practices (putting things inside your vagina or making small cuts around it)? Choose all that apply, you may choose more than one:
VP6801A	What do you think it might happen if you stop the vaginal practices (putting things inside your vagina or making small cuts around it)? Choose all that apply, you may choose more than one:: I will feel dirty	1
	0	=	No
	1	=	Yes

VP6801B	What do you think it might happen if you stop the vaginal practices (putting things inside your vagina or making small cuts around it)? Choose all that apply, you may choose more than one:: I will feel uncomfortable	1
	0	=	No
	1	=	Yes

VP6801C	What do you think it might happen if you stop the vaginal practices (putting things inside your vagina or making small cuts around it)? Choose all that apply, you may choose more than one:: I will get a discharge	1
	0	=	No
	1	=	Yes

VP6801D	What do you think it might happen if you stop the vaginal practices (putting things inside your vagina or making small cuts around it)? Choose all that apply, you may choose more than one:: I will get an odor	1
	0	=	No
	1	=	Yes

VP6801E	What do you think it might happen if you stop the vaginal practices (putting things inside your vagina or making small cuts around it)? Choose all that apply, you may choose more than one:: My partner will not like it	1
	0	=	No
	1	=	Yes

VP6801F	What do you think it might happen if you stop the vaginal practices (putting things inside your vagina or making small cuts around it)? Choose all that apply, you may choose more than one:: My vagina will be healthier	1
	0	=	No
	1	=	Yes


Q115.	What would you do if you hear that vaginal practices (putting things inside your vagina or making small cuts around it) are bad for you?
VP6901	What would you do if you hear that vaginal practices (putting things inside your vagina or making small cuts around it) are bad for you?	1
	1	=	I would stop doing them
	2	=	I would do less
	3	=	I would use different products
	4	=	Vaginal practices are not bad and I would not change anything

Q116.	How easy or difficult do you think it will be to stop doing vaginal practices (putting things inside your vagina or making small cuts around it
VP7001	How easy or difficult do you think it will be to stop doing vaginal practices (putting things inside your vagina or making small cuts around it	1
	1	=	It will difficult since I have done it for so long
	2	=	It will be very easy if they are bad for me.
	3	=	Some practices will be easy to discontinue some not.

Q117.	Which type or vaginal practices would be the least willing to change?
VP7101	Which type or vaginal practices would be the least willing to change?	2
	1	=	Putting my  fingers inside my vagina
	2	=	Putting water inside my vagina
	3	=	Putting soap (lifeboy or other) inside my vagina
	4	=	Putting Cloth,paper, wipe or sponge inside my vagina
	5	=	PuttingHerbs or flowers from the garden inside my vagina
	6	=	Putting Herbs or flowers - traditional medicines inside my vagina
	7	=	Putting Lager Beer inside my vagina
	8	=	Putting Ripe lemon inside my vagina
	9	=	Putting Vinegar inside my vagina
	10	=	Putting Salt inside my vagina
	11	=	Making Small cuts around the vagina
